# Neutrophil Extracellular Traps (NETs) and Hypercoagulability in Plasma Cell Dyscrasias—Is This Phenomenon Worthy of Exploration?

**DOI:** 10.3390/jcm10225243

**Published:** 2021-11-11

**Authors:** Olga Ciepiela, Milena Małecka-Giełdowska, Emilia Czyżewska

**Affiliations:** Department of Laboratory Medicine, Medical University of Warsaw, Banacha 1a, 02-091 Warsaw, Poland; milena.malecka@wum.edu.pl (M.M.-G.); emilia.czyzewska@wum.edu.pl (E.C.)

**Keywords:** plasma cell dyscrasias, multiply myeloma, hypercoagulability, neutrophil extracellular traps (NETs)

## Abstract

Plasma cell dyscrasias (PCDs) are neoplastic diseases derived from plasma cells. Patients suffering from PCDs are at high risk of hypercoagulability and thrombosis. These conditions are associated with disease-related factors, patient-related factors, or the use of immunomodulatory drugs. As PCDs belong to neoplastic diseases, some other factors related to the cancer-associated hypercoagulability state in the course of PCDs are also considered. One of the weakest issues studied in PCDs is the procoagulant activity of neutrophil extracellular traps (NETs). NETs are web-like structures released from neutrophils in response to different stimuli. These structures are made of deoxyribonucleic acid (DNA) and bactericidal proteins, such as histones, myeloperoxidase, neutrophil elastase, and over 300 other proteins, which are primarily stored in neutrophil granules. NETs immobilize, inactivate the pathogens, and expose them to specialized cells of immune response. Despite their pivotal role in innate immunity, they contribute to the development and exacerbation of autoimmune diseases, trigger inflammatory response, or even facilitate the formation of cancer metastases. NETs were also found to induce activity of coagulation and are considered one of the most important factors inducing thrombosis. Here, we summarize how PCDs influence the release of NETs, and hypothesize whether NETs contribute to hypercoagulability in PCDs patients.

## 1. Introduction

Plasma cells are non-dividing and fully differentiated cells arising from the B cell line. Their primary role is to synthesize antibodies as a part of the humoral immune response. A single plasma cell produces molecules of immunoglobulin in a specific class. In the course of humoral response, polyclonal plasma cells produce polyclonal immunoglobulins with different heavy and light chains. As a cellular part of the hematopoietic system, plasma cells under specific circumstances may transform into neoplastic cells, with the potential for proliferation and the development of cancers. Neoplasms derived from plasma cells are known as plasma cell dyscrasias (PCDs) [1]. 

PCDs consist of three more specified subgroups of disease: multiple myeloma, solitary plasmacytoma, and monoclonal immunoglobulin deposition disease (AL amyloidosis). Multiple myeloma accounts for 1–1.8% of all cancers, and, together with diffuse large B-cell lymphoma (DLBCL), is the second most common hematological malignancy with an estimated incidence in Europe of 4.5–6.0 cases per 100,000 per year [2,3]. A pre-myeloma condition, called monoclonal gammopathy of undetermined significance (MGUS), is recognized in 3–4% of the adult population >50 years old (y.o.), and even in over 5% of adults older than 70. Patients diagnosed with MGUS do not present symptoms of organ damage typical in multiple myeloma; nevertheless, monoclonal protein of less than 30 g/L can be found in their serum [2,4].

To diagnose plasma cell dyscrasia, clonal plasma cells should be found in the bone marrow using immunophenotyping or immunohistochemistry staining. It has to be underlined that not all types of plasma cell dyscrasias release monoclonal immunoglobulins; thus, detection of monoclonal protein in serum electrophoresis and immunofixation is not necessary for final diagnosis. However, a final diagnosis of secretory or non-secretory multiple myeloma has to be stated. As a result of the clonal proliferation of plasma cells, several organ injuries may appear in a patient. There are established criteria for organ injury in the course of multiple myeloma (SLiM CRAB), and they include serum **C**alcium concentration >2.75 mmol/L, **R**enal insufficiency with creatinine serum concentration >177 µmol/L, **A**nemia with hemoglobin concentration <10 g/dL, osteolytic cluster in the **B**one, at least **S**ixty percent (60%) of clonal plasma cells in the bone marrow, ratio of involved and uninvolved **Li**ght chains of immunoglobulins of at least 100, and at least two focal lesions in **M**agnetic resonance [2].

Delayed treatment may result in irreversible organ damage. Renal insufficiency that arises in the course of plasma cell dyscrasias (as stated in SLiM CRAB criteria) results from various pathologies:

– Accumulation of light immunoglobulin chains in distal tubules, forming obstructive casts;
– Direct toxicity of light chains to proximal tubules, where they are endocytosed and induce necrosis of tubule cells; and
– Hypercalcemia, decreasing renal concentrating ability [5].

Increased serum protein concentration that occurs in plasma cell dyscrasias and contributes to renal insufficiency entails the risk of developing hyperviscosity syndrome. This is triggered by hyperproteinemia, causing an impairment in blood flow in the microvasculature. Along with neurological manifestation and retinal changes caused by insufficient blood flow, hyperviscosity may induce vein thrombosis [4]. 

## 2. Hypercoagulability in Plasma Cell Dyscrasias 

In general, patients suffering from plasma cell dyscrasias, especially multiple myeloma, are at increased risk of hypercoagulability and venous thromboembolism development [6,7,8,9,10,11]. As many as 10% of patients suffering from multiple myeloma are affected by venous thrombotic events (VTE), which are found to be associated with the risk of pre-term death [6]. This phenomenon is not only dependent on hyperviscosity; several studies have shown that thrombotic events are associated with disease-related factors (including prothrombic properties of monoclonal proteins or enhanced production of prothrombic agents), patient-related factors (such as obesity or impaired motility), or the use of immunomodulatory drugs [2]. 

**Figure 1:**
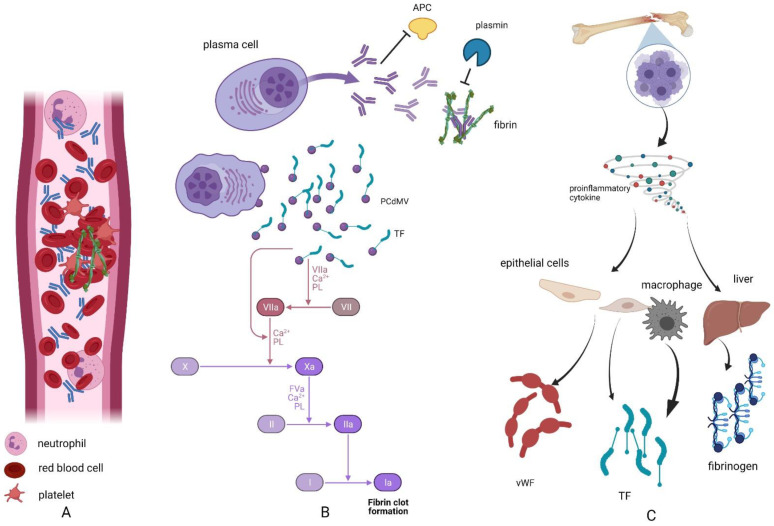
Mechanisms inducing hypercoagulability in plasma cell dyscrasias: (**A**) Extent of immunoglobulins in blood leads to hyperviscosity and a slowdown in blood flow, which in turn leads to platelet activation and the activation of coagulation. (**B**) Procoagulant effect of plasma cells is also associated with direct effect of monoclonal immunoglobulin on natural inhibitors of coagulation, leading to resistance to active protein C. Furthermore, plasma cells were found to release microvesicles baring tissue factor, which activates the extrinsic pathway of coagulation. In addition, monoclonal immunoglobulins attached to the thrombi block fibrinolysis. (**C**) Proliferation of monoclonal plasma cells in the bone marrow leads to overproduction of proinflammatory cytokines (i.e., interleukin 6 (IL-6), Tumor Necrosis Factor α (TNF-α)), which in turn stimulates production of procoagulation factors (fibrinogen in the liver, tissue factor in macrophages, and epithelial cells or von Willebrand factor in epithelial cells). APC, active protein C; vWF, von Willebrand factor; TF, tissue factor; PCdMV, plasma cell-derived microvesicles. The figure was generated using BioRender.com (22 September 2021).

Disease-related factors that are associated with increased risk of VTE include the procoagulant effect of monoclonal protein itself. It has been reported that monoclonal immunoglobulins not only interfere with fibrin formation but also delay fibrinolysis [10,12]. It has been shown that mechanisms influencing coagulation in patients with plasma cell dyscrasias are acquired resistance to protein C, which is found in 6–23% patients, as well as an interference of monoclonal protein in clot fibrinolysis [10,13]. Moreover, products of neoplastic plasma cells appear to have lupus anticoagulant-like activity, and autoantibodies decrease the level of antithrombin and proteins S and C, which are important natural anticoagulants [10,12]. Factors that are unrelated to the presence of monoclonal proteins are: increased concentration of inflammatory cytokines (interleukin 6 (IL-6); tumor necrosis factor-α (TNF-α); vascular endothelial growth factor (VEGF)), which in turn increases the production of procoagulant von Willebrand factor (vWF), fibrinogen, or tissue factor (TF) [11,12]. Finally, it has been reported that myeloma cells release specific microparticles that express tissue factor (TF) and promote hypercoagulability [7,10,11,14] (Figure 1).

Detailed mechanisms of the procoagulant action of immunomodulatory drugs including thalidomide, lenalidomide, and pomalidomide remain unknown [8]; however, observational studies report that the use of these medications in mono- or multidrug-therapy may increase the risk of VTE by up to 30% [6]. Nonetheless, the simultaneous use of bortezomib, a protease inhibitor that acts hemostatically, counterbalances the prothrombic effect of immunomodulatory drugs [2].

## 3. Neutrophil Extracellular Traps and Coagulation

As multiple myeloma belongs to neoplastic diseases, some other factors related to cancer-associated hypercoagulability in the course of plasma cell dyscrasias are also considered. One of the weakest issues in studies related to plasma cell dyscrasias is the procoagulant activity of NETs. NETs are web-like structures released from neutrophils in response to different stimuli, either physiological or non-physiological. To date, more than 870 agents have been identified to trigger the release of NETs, including phorbol 12-myristate 13-acetate (PMA), H_2_O_2_, growth factors, platelets, calcium, glucose, bacterial- or fungal-derived products, and even bacteria and fungi [15]. 

The release of NETs is an active mechanism belonging to the wide armamentarium of innate immune response. These structures are made of DNA and bactericidal proteins, such as histones, myeloperoxidase, neutrophil elastase, and over 300 other proteins, which are primarily stored in neutrophil granules. In the course of NETs release, there is a decondensation of chromatin, citrullination of histones by peptidyl arginine deiminase 4 (PAD4)—a calcium-dependent enzyme—degradation of nuclear membrane, transfer of neutrophil elastase to the nucleus, integration of nuclear content with substances stored within cytoplasmatic granules, and, finally, the rupture of plasma membrane and active eruption of NETs into extracellular space [16,17,18,19]. There, NETs immobilize, inactivate pathogens, and expose them to specialized cells of the immune response. Despite their pivotal role in innate immunity, they contribute to the development and exacerbation of autoimmune diseases, and, being a source of autoantigens, trigger inflammatory response or even facilitate the formation of cancer metastases [17,18,20]. NETs were also found to induce activity of coagulation and are considered one of the most important factors inducing thrombosis in cancer patients suffering from cancer or generalized inflammatory diseases [14,18,19,20,21,22,23,24,25,26,27,28,29].

The way in which NETs promote coagulation is complex and involves the activation of either platelets or plasma coagulation factors [18]. The interaction between platelets and neutrophils/NETs is mutual. It has been shown that platelets and platelet-derived factors (i.e., high mobility group box 1 protein (HGMB1); P-selectin; complex of α_IIb_β_3_ integrin and choline transporter-like protein 2, α_IIb_β_3_-CTL2) can induce neutrophils to release NETs [14,15,18,27]. This interaction may be associated with binding of platelets to neutrophils via glycoprotein Ib (GPIb) [30]. In contrast, NETs and their components activate platelets by histones H3 and H4 and histone/DNA complexes [14,18,22,29] through stimulation of Toll-like receptors TLR2 and TLR4 on the surface of platelets [30]. NETs were found to induce aggregation and activation of platelets, which was confirmed by the release of adenosine triphosphate (ATP) and adenosine diphosphate (ADP) from platelets and an increase in surface expression of CD62L and phosphatidylserine [31]. Importantly, no DNA or histones are involved in platelets’ aggregation in vitro, but cathepsin G is found in the structure of NETs and the phosphorylation of Syk tyrosin kinase in platelets [32]. Moreover, NETs become a scaffold for platelets and red blood cells on which they can aggregate and, together with fibrin, they form a thrombus [19,21,28,29,33]. In their study, Fuchs et al. proved that a NETs-based thrombus may be stable even if fibrin is removed from the scaffold, suggesting that platelets–red blood cell–DNA binding is strong enough to form solid thrombi [33]. Further, it is suggested that thrombus with involved NETs is generated as a result of the interaction between citrullinated H3 from NETs and the von Willebrand factor [30]. 

**Figure 2:**
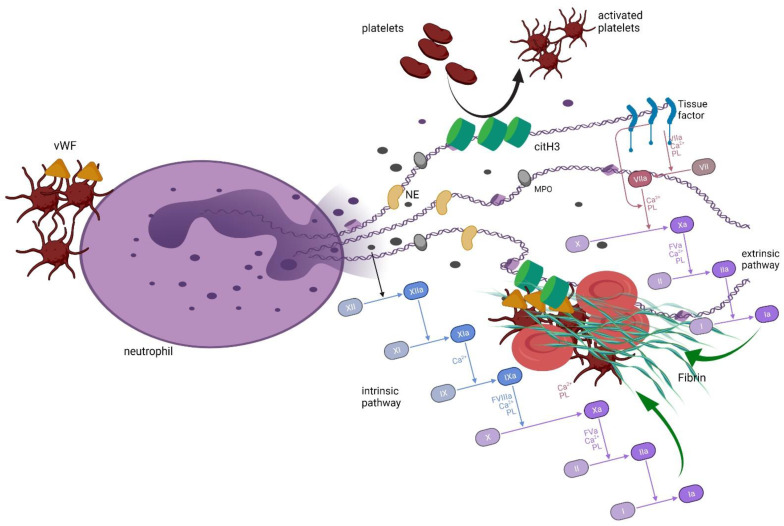
The role of NETs in inducing coagulation and formation of thrombi. Activated platelets may induce neutrophils to release NETs. Citrullinated histones activate platelets, which leads to activation of coagulation. Moreover, DNA from NETs become a scaffold for thrombus generation by attaching red blood cells and activated platelets. Negative charge of DNA threads activate FXII, inducing intrinsic pathway of coagulation. Tissue factor that is exposed in the NETs structure activates extrinsic pathway of coagulation. NETs, neutrophil extracellular traps; FXII, coagulation factor XII; NE, neutrophil elastase; MPO, myeloperoxidase; vWF, von Willebrand factor; citH3, citrullinated histone 3. The figure was generated using BioRender.com (22 September 2021).

Coagulation may be also triggered by NETs in the platelet-independent pathway. It has been shown that NETs themselves can induce and activate coagulation factor XII (FXII), simultaneously inhibiting the tissue factor pathway inhibitor (TFPI) by serine proteases, together promoting coagulation [22,23,28,30]. Furthermore, negatively charged DNA found in the NETs’ composition was found as a factor that activated the intrinsic pathway of coagulation [31,34,35]. Interestingly, the interaction between FXII and NETs is not limited to only a one-way effect; Stavrou et al. found that FXII together with zinc (Zn^2+^) promoted citrullination of histone H3, and the pathway that involves the binding of FXII to urokinase plasminogen activator receptor (uPAR) triggered DNA release from the cell [36]. Moreover, both tissue factor (TF) and von Willebrand factor have been observed in the NETs composition, together with coagulation factor X, prothrombin, and fibrinogen, leading to the suspicion that NETs trigger coagulation by affecting either intrinsic or extrinsic pathways of coagulation [18,19,22]. Direct evidence of NETs’ engagement in thrombi generation is also provided: immunohistochemistry staining of thrombi revealed colocalization of histones, neutrophil elastase, myeloperoxidase, DNA, citrullinated H3 histone, and citrullinated H3 in either venous or arterial thrombi [19,28]. Furthermore, thrombi were found to lyse faster in the presence of DNAse and tissue-type plasminogen activator (tPA) compared to tPA alone, supporting the observation of NETs’ involvement in thrombus generation [35,37] (Figure 2).

## 4. Neutrophil Extracellular Traps in Plasma Cell Dyscrasias

The knowledge regarding NETs’ release in the course of plasma cell dyscrasias is very poor. Even less is known about the potential pro-coagulation effect of NETs in neoplasms derived from plasma cells. Using a mouse model, Li et al. found that neutrophils isolated from animals with multiple myeloma were able to release more NETs under the influence of PMA than neutrophils isolated from healthy mice. Such an effect was observed in mice into which different myeloma cells (DP42 and 5TGM1) were injected [38]. In vitro studies by Fagerhol et al. reveal that patients suffering from multiple myeloma have a higher concentration of S100A12/calprotectin complexes than healthy subjects, and S100A12/calprotectin complexes in this study were used as markers of NET release [39]. However, it should be taken into consideration that the aforementioned complexes poorly correlated with calprotectin alone, which was previously confirmed to be a component of NETs. Moreover, S100A12 and calprotectin are both markers of inflammation, and their plasma concentration increase may be indicative of an inflammatory state and not necessarily associated with NETs’ release [40]. Thus, these observations need further investigation and confirmation. Li et al. also found that myeloma cells may induce NETs’ release; they performed a study in which mice neutrophils were incubated with multiple myeloma cells (DP42 and 5TGM1) or a medium from their culture. Both these agents induced the release of murine NETs and citrullination of histones, which proves that multiple myeloma cells and their products act through activation of PAD4. Next, they inhibited PAD4 to examine whether neoplastic cells could still trigger NETs release and found a lack of stimulation to NETs by these cells. A similar effect was found when human neutrophils were first incubated with multiple myeloma cell lines and later with a PAD4 inhibitor. Surprisingly, they also found that the progression of a cancer was delayed by using a PAD4 inhibitor (BMS-P5) in a multiple myeloma mice model in vivo [41]. These observations are the first and the only ones in the field of NET induction in multiple myeloma subjects and still do not explain the factors that are associated with myeloma cells contributing to neutrophils’ activation to release NETs. The authors underline that targeting one of the NETs’ mechanisms may be beneficial in the treatment of multiple myeloma [41]. 

The first study in which a potential effect between NETs and coagulation in multiple myeloma was investigated was performed by Lauw et al., and their results were only presented as an abstract at the 58th American Society of Hematology Annual Meeting. The authors had indeed found increased amounts of nucleosomes in the plasma of multiple myeloma patients, but they did not find any correlation between the concentration of nucleosomes and venous thromboembolism events. However, they rightly pointed out that the NETs were not the only source of nucleosomes but that they came mainly from dead cells, which are elevated in cancer patients [42].

A review of the literature in the field reveals that studies performed in plasma cell dyscrasias are limited almost exclusively to multiple myeloma, leaving scientific questions regarding these processes (hypercoagulation and NETs release) in other plasma cell dyscrasias unanswered. Only one report was found regarding NETs’ release under the influence of amyloid fibers, although not with fibers formed of immunoglobulin light chains but artificially constructed with yeast prion, A25T, associated with oculoleptomeningeal amyloidosis, and α-synuclein associated with Parkinson disease [43].

## 5. Summary

**Figure 3:**
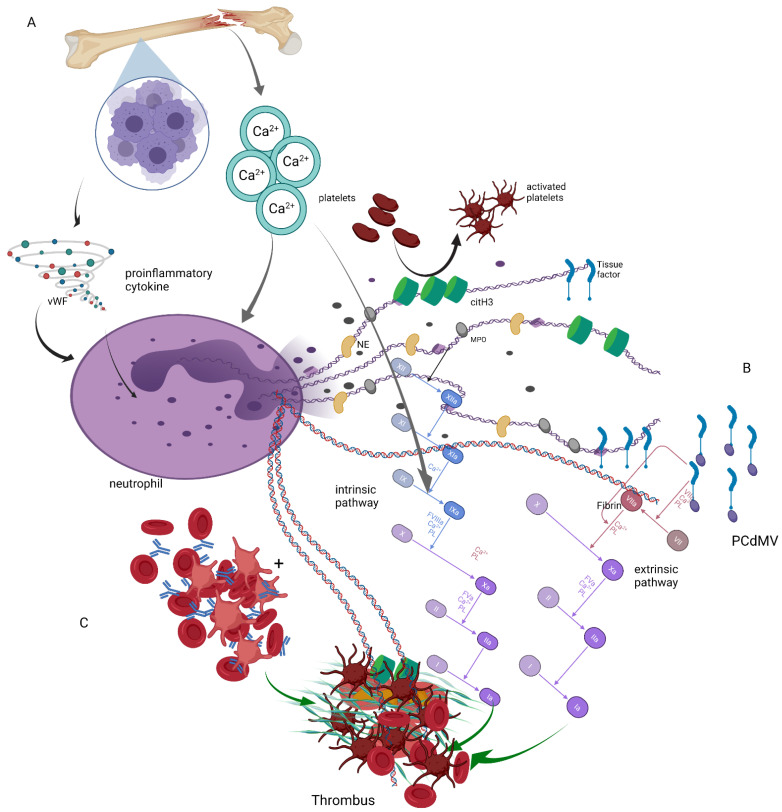
Joint effects of NETs and plasma cell dyscrasias on thrombus formation: (**A**) Proinflammatory cytokines released from bone marrow due to neoplastic plasma cells proliferation trigger NETs’ release, and similar effect may be observed under the influence of calcium over-released from plasma cell-lysed bones. Moreover, calcium contributes to activation of coagulation cascade. (**B**) Tissue factor either trapped in the NETs or bound to plasma cell-derived microvesicles (PCdMV) activates the extrinsic pathway of coagulation. (**C**) Red blood cells and platelet aggregates formed due to blood hyperviscosity in the course of PCDs may be trapped into NETs and form DNA-based thrombus. NE, neutrophil elastase; MPO, myeloperoxidase; vWF, von Willebrand factor; citH3, citrullinated histone 3, PCdMV, plasma cell derived microvesicles. The figure was made using BioRender.com (7 November 2021).

As stated, there is irrefutable evidence that NETs contribute to hypercoagulability in cancer patients. Moreover, the procoagulant effect of multiple myeloma itself and its treatment has also been proven. However, there is no proven information regarding the potential synergistic effects of both neoplasm and NETs on hypercoagulability in plasma cell dyscrasias. As mentioned above, both NETs and PCDs may lead to hypercoagulability. Moreover, there are some joint mechanisms triggered by both NETs and PCDs that lead to thrombus generation, including extrinsic coagulation pathway activation, neutrophil stimulation by proinflammatory cytokines, or generation of platelets and red blood cells aggregates that may form thrombus. Furthermore, it has to be mentioned that a typical PCDs increase in plasma calcium may also contribute to the generation of NETs and the activation of coagulation (Figure 3). 

Shi et al. showed that calcium is necessary to induce PAD4-mediated histone citrullination [44], while other researchers reported that calcium is crucial for the production of mitochondrial reactive oxygen species (ROS) and the activation of nicotinamide adenine dinucleotide phosphate (NADPH) oxidase—an enzyme involved in NETs release [45]. Additionally, calcium overload and calcium-based crystals may, independently from ionized calcium influx into the cell, induce NETs release, as shown by Leppkes et al. in a study assessing NETs’ release after neutrophil stimulation by calcium carbonate crystals from pancreatic juice [46].

All things considered, the coagulation stimulating effect of NETs in plasma cell dyscrasias seems to be an unexplored area of research. To date, no studies have been performed in AL amyloidosis or MGUS—a plasma cell dyscrasia with the highest frequency in the population in general. Thus, developing this knowledge may shed new light upon the pathogenesis of thrombi generation in multiple myeloma and other monoclonal gammopathies; this approach may, in the future, also contribute to the development of a more efficient thromboprophylaxis in this group of patients.
